# Differing myocardial response to a single session of hemodialysis in end-stage renal disease with and without type 2 diabetes mellitus and coronary artery disease

**DOI:** 10.1186/1476-7120-4-9

**Published:** 2006-02-02

**Authors:** Satish Chandra Govind, Simin Roumina, Lars-Åke Brodin, Jacek Nowak, Saligrama Srinivasiah Ramesh, Samir Kanti Saha

**Affiliations:** 1 Bhagwan Mahavir Jain Heart Center, Bangalore, India; 2 Karolinska University Hospital at Huddinge, Stockholm, Sweden

## Abstract

**Background:**

Though hemodialysis (HD) acutely improves cardiac function, the impact of background diseases like coronary artery disease (CAD) and Type 2 diabetes (DM) in the setting of end-stage renal disease (ESRD) is not known. Tissue velocity echocardiography (TVE) offers a fast choice to follow changes in myocardial function after HD in ESRD with concomitant DM and /or CAD.

**Methods:**

46 subjects (17 with ESRD, Group 1; 15 with DM, Group 2; 14 with DM+CAD, Group 3) underwent standard and TVE prior to and shortly after HD. Besides standard Doppler variables, regional myocardial systolic and diastolic velocities, as well as systolic strain rate were post processed.

**Results:**

Compared with pre-HD, post-HD body weight (kg) significantly decreased in all the three groups (51 ± 9 vs. 48 ± 8, 62 ± 10 vs.59 ± 10, and 61 ± 9 vs. 58 ± 9 respectively; all p < 0.01). Left ventricular end diastolic dimensions (mm) also decreased post- HD (46 ± 5 vs. 42 ± 7, 53 ± 7 vs. 50 ± 7, 51 ± 7 vs. 47 ± 8 respectively; all p < 0.01). Regional longitudinal peak systolic velocity in septum (cm/s) significantly increased post-HD in Group 1(5.7 ± 1.6 vs. 7.2 ± 2.3; p < 0.001) while remained unchanged in the other two groups. Similar trends were noted in other left ventricular walls. When the myocardial velocities (cm/s) were computed globally, the improvement was seen only in Group 1 (6.3 ± 1.5 vs. 7.9 ± 2.0; p < 0.001). Global early regional diastolic velocity (cm/s) improved in Group 1, remained unchanged in Group 2, while significantly decreased in Group 3(-5.9 ± 1.3 vs. -4.1 ± 1.8; p < 0.01). Global systolic strain rate (1/sec) increased in the first 2 Groups but remained unchanged (-0.87 ± 0.4 vs. -0.94 ± 0.3; p = ns) in Group 3.

**Conclusion:**

A single HD session improves LV function only in ESRD without coexistent DM and/or CAD. The present data suggest that not only dialysis-dependent changes in loading conditions but also co-existent background diseases determine the myocardial response to HD.

## Background

Patients with Type 2 diabetes mellitus (DM) of long duration frequently suffer from end-stage renal disease (ESRD) and a great majority of them also have coexistent coronary artery disease (CAD) [1,2]. Patients with primary ESRD are at risk of having a coronary event as well and often die of cardiac causes [3-5]. Detailed mechanisms behind cardiac deaths have been relatively well described, but exact changes in the regional contractile behaviors of the left ventricle (LV) are not clearly established in ESRD, particularly during on-going renal replacement therapy such as hemodialysis (HD), a procedure destined to temporarily remove excess fluids and toxins.

Though it has been already demonstrated in patients undergoing HD that concomitant changes in the TVE variables are load dependant [6-9], it is however still not known whether ESRD with coexistent DM and/or CAD, i.e., conditions in which regional myocardial systolic and diastolic velocities are already diminished [10-13], have differing myocardial response following dialysis-dependent acute changes in preload.

Therefore, in this study, we have tested the hypothesis that longitudinal myocardial functions as assessed by TVE in response to HD are quantitatively different in differing clinical settings irrespective of similar degree of changes in loading conditions.

## Methods

### Study design

The number of study subjects was 46. Patients with primary myocardial disease, significant pericardial disease, significant valvular disease or arrhythmias were excluded. Diagnoses of the illnesses were defined by standard criteria, medication and treatment history and from the hospital database. Diagnosis of CAD was on the basis of clinical history, findings of definite ECG changes of ischemia or previous myocardial infarction, echocardiographic findings of regional wall motion abnormality (n = 9) and those having angiographic evidence of significant coronary artery disease (n = 5). All biochemical analyses were done prior to HD, using a standard spectrophotometer.

The Institutional Review Board of Bhagwan Mahavir Jain Heart Centre, Bangalore, India, approved the study protocol. All the study subjects gave their informed consent to participate.

### Standard echocardiography protocol

Echocardiography was performed using a VIVID 5 equipment (GE Vingmed, Horten, Norway) with a preinstalled Echopac 6.3.4 software program. A 2.5 MHz probe was used for image acquisition. The images were acquired in parasternal long- and short-axis and in apical 2-, 3-, and 4- chamber projections. LV dimensions and wall thicknesses were measured by M-mode in the parasternal long-axis views. Mitral inflow velocities were measured by conventional pulsed wave Doppler, by positioning the sample volume at the level of the tips of mitral leaflets in the apical 4-chamber views. The peak early (E) and late (A) diastolic velocities, and E/A ratio were measured. 2-D LV ejection fraction was measured by modified Simpson's approach. All 2-D and M-mode measurements were made according to the American Society of Echocardiography guidelines.

### Tissue velocity echocardiography

**Figure 1:**
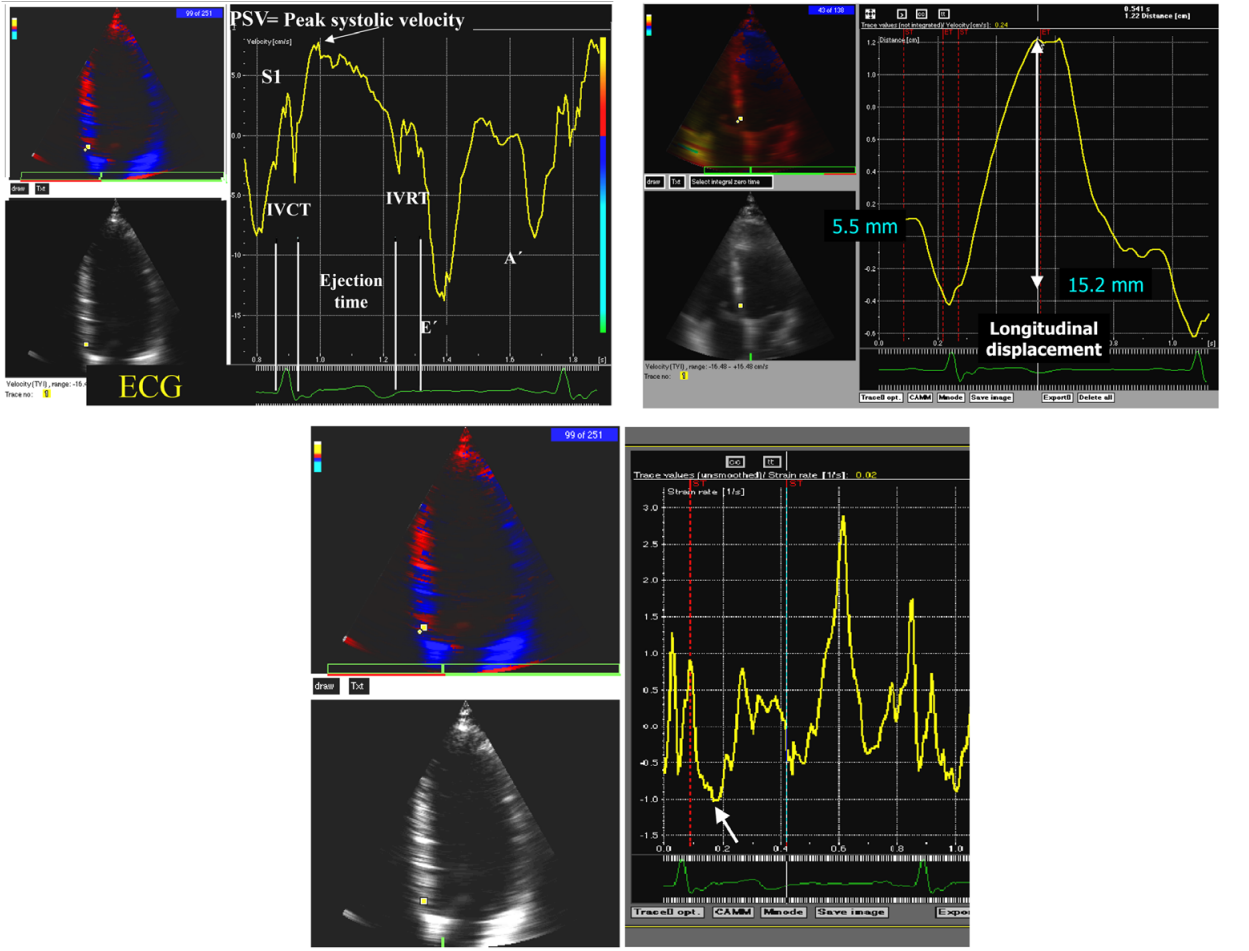
*Upper left:* A typical color-Doppler velocity profile obtained by positioning the sample volume in the septum base. S1 = isovolumic contraction velocity in the first positive peak, PSV = peak systolic velocity. IVCT and IVRT respectively are isovolumic contraction and relaxation times, E'and A'are respectively early and late diastolic velocities. The velocity profile is accompanied by an ECG signal. *Upper right*. A systolic displacement image, obtained by integration of the respective velocity profile during systole that provides amplitude of total displacement (usually ≥ 12 mm in healthy individuals). Lower panel. Strain rate imaging in which the white arrow indicates peak systolic strain rate obtained by spatial differential of velocities at two points divided by the distance between them. In our laboratory the insonation distance of approximately 15 mm provides a favorable signal-to-noise ratio [27].

Digitally stored cine loops during three consecutive heart cycles were analysed offline using Echopac 6.3.0 software (GE Vingmed, Horten, Norway). The LV long-axis regional function was assessed from the apical views from which four basal segments (septum, lateral, inferior, and anterior) were analysed. The cursor was placed in the basal segment so as to exclude the mitral annulus. A typical velocity profile could be obtained by positioning the sample volume in any of the basal segments. Peak systolic (PSV), early diastolic (E'), and late diastolic (A') myocardial velocities (cm/sec) were measured at the peaks of the respective waves on the myocardial velocity curves in the individual LV walls (Figure 1). The corresponding global variables were measured by taking average of the 4 LV bases. The LV short-axis systolic and diastolic functions were assessed from the parasternal short-axis images, from which only the mid posterior segments (parasternal short-axis image) were analysed in order to compute radial data. Isovolumic contraction (IVCT) and relaxation (IVRT) times as well as ejection (ET) times were also measured according to methods described by Lind et al [14]. Filling time (FT) was measured from the end of IVRT to the beginning of IVCT. The myocardial wall displacement (mm) in the long- and short-axis was obtained by automated temporal integration of the velocity profile during systole (Figure 1). E/E' was measured from the septal and lateral annulus from the apical 4-chamber projection. LV myocardial performance index, Tei index [15], was defined as the sum of IVCT and IVRT divided by ejection time using the color Doppler. Global Tei index was calculated by taking the average data from 4 LV bases. Images were stored at an average frame rate of 130 s^-1 ^for post processing.

### Strain rate imaging

Strain represents deformation of an object (in this case the myocardial wall) and is often expressed in percentage of change from end-diastolic dimension [16]. Positive strain represents lengthening or stretching while negative strain is shortening or compression. Strain rate (SR) is the instantaneous strain per unit time (cm/s/cm, or 1/sec) and has the same direction as the strain, i.e. negative strain rate during shortening and positive strain during lengthening (Figure 1, lower panel). Strain rate therefore represents velocity of regional myocardial deformation, thereby also reflecting myocardial contraction when measured at peak systole. To calculate strain rate the area of interest along the sample scale was chosen to be ≈ 15 mm since this appears to provide the best signal to noise ratio. The longitudinal basal, mid and apical segments of septum, lateral, inferior and anterior walls were averaged to assess global strain rate. Radial strain rate was calculated from the LV posterior wall in the short axis projection. Similar frame rates used for velocity data were also used to measure strain rate.

### Hemodialysis

All patients were on maintenance HD, done twice weekly. Duration of maintenance HD varied from 10 months to 7 years. Echocardiography was done within 60 minutes of pre- and postdialysis. Hemodialysis was performed using Fresemins-76 dialyser with dialysate flow rate of 500 ml/min. Sodium bicarbonate dialysis was done with dialysate flow of 500 ml/min and an average blood flow of 250 ml/min.

### Coronary angiography

Coronary angiographies were done using the Judkins technique. Significant stenosis was defined as >70 % intra-luminal obstruction either visually or quantitatively by coronary angiography.

### Statistical methods

Data are expressed as mean ± SD. One-way ANOVA followed by Scheffe's test was performed to compare the differences among the three groups. Paired t test was performed to compare pre-and post-HD data within groups. A PC-based version of STATISTICA version 6.0 was used for data analyses. Intra-observer variation was similar to our previous study [11]. All continuous variables are expressed as mean ± SD. A p value of <0.05 was considered statistically significant.

## Results

Of the study subjects 31 were males and 15 females. The age (years) of the subjects respectively in the 3 groups were 40 ± 16, 57 ± 7, and 56 ± 9 (p < 0.05 for Group 1 vs. others). The study subjects were non-consecutively selected and divided as Group 1: ESRD due to primary renal disease (n = 17), Group 2: ESRD+DM (n = 15), and Group 3: ESRD+DM+CAD (n = 14). Patients with primary ESRD had polycystic kidney disease, IgA nephropathy, focal segmental glomerulosclerosis, or crescent glomerulonephritis. Remaining clinical characteristics of the study population are given in Table 1.

**Table 1:** Clinical characteristics of the study subjects

Average age of the patients (years)	51 ± 14
Average body weight (kg)	58 ± 6
Number of male/female pts	31/15
Average systolic BP (mmHg)	146 ± 11
Average diastolic BP (mmHg)	87 ± 8
Duration of DM	14 ± 6 years
Number of DM subjects receiving insulin	9
Number of patients receiving insulin plus OHD	37

OHD = Oral hypoglycaemic drugs; DM= Type 2 diabetes mellitus

Table 2 provides the biochemical data that show plasma glucose being the lowest and serum HDL highest in the ESRD group while serum creatinine was the lowest in ESRD+DM+CAD group (all relevant p < 0.05). Blood hemoglobin levels were equally low in all the groups. Elevated glucose level was present in the DM groups while serum cholesterol and triglycerides were non-significantly elevated only in the ESRD+DM+CAD group.

**Table 2:** Biochemical data in the three study groups at pre-HD

	**ESRD Group 1 (n = 17)**	**ESRD+DM Group 2 (n = 15)**	**ESRD+DM+CAD Group 3 (n = 14)**
Blood Urea Nitrogen (mg/dl)	152.33 ± 81.94	112.29 ± 24.64	107.07 ± 32.58
S.Creatinine (mg/dl)	9.42 ± 2.53	9.0 ± 1.42	7.46 ± 1.94 *
Hemoglobin (mg/dl)	7.97 ± 1.13	8.73 ± 0.87	8.05 ± 1.33
S.Glucose (mg/l)	101.00 ± 9.44^†^	169.95 ± 74.06	161.10 ± 67
S.Sodium (mmol/l)	139.89 ± 5.19	131.72 ± 35.31	140.15 ± 3.29
S.Potassium (mmol/l)	5.41 ± 0.70	5.27 ± 0.94	5.64 ± 0.74
S.Calcium (mg/dl)	8.24 ± 0.79	7.85 ± 0.72	8.02 ± 0.58
S.Phosphate (mg/dl)	5.31 ± 1.68	5.22 ± 1.15	4.41 ± 1.02
S. Cholesterol (mg/dl)	176.06 ± 19.90	183.85 ± 35.64	238.25 ± 59.99
S.Triglycerides (mg/dl)	148.13 ± 24.26	155.77 ± 29.97	172.25 ± 29.59
S.HDL (mg/dl)	45.94 ± 5.97^‡^	36.77 ± 6.64	40.67 ± 4.25
S.LDL (mg/dl)	84.06 ± 15.46	95.77 ± 27.06	108.00 ± 20.63

Data Data are means ± SD. Group comparisons were made using one way ANOVA followed by Scheffe's test. * p < 0.05 Group 1 vs. Group 3; ^† ^p < 0.01 Group 1 vs. Group 2 and Group 3; ^‡ ^p < 0.01 Group 1 vs. Group 2. HDL = high density lipoprotein; LDL = low density lipoprotein. S = Serum

Table 3 shows that there were no changes in the heart rate (R-R interval) and systolic and diastolic blood pressures at pre- or post-HD, but body weight decreased significantly in all the groups at post-HD. LV hypertrophy at predialysis was seen in 7 ESRD, 10 ESRD+DM, and 12 ESRD+DM+CAD subjects, respectively, with no significant change at post-HD. 2-D obtained LV ejection fraction (>40% in all the 3 groups) did not show any significant changes within the groups at pre- and post-HD. LV internal dimensions showed significant decrease in all the groups post- HD. The velocity of early diastolic filling (E) decreased in ESRD and ESRD+DM+CAD groups while deceleration time (DT) increased in ESRD+DM+CAD group after dialysis. E/A ratio obtained from the standard transmitral Doppler was lower in all the groups at post HD (all p < 0.05).

**Table 3:** 2-D Echocardiography data, body weight and blood pressure measurements pre- and post-hemodialysis

	**ESRD (Group 1)**		**ESRD+DM (Group 2)**		**ESRD+DM+CAD (Group 3)**	
	**PRE**	**POST**	**PRE**	**POST**	**PRE**	**POST**
Weight (kg)	51 ± 9	48 ± 8*	62 ± 10	59 ± 10*	61 ± 9	58 ± 9*
R-R interval(ms)	705 ± 137	683 ± 207	707 ± 122	683 ± 134	701 ± 92	714 ± 118
Systolic BP (mm of Hg)	145 ± 26	136 ± 20	147 ± 11	140 ± 16	147 ± 21	139 ± 14
Diastolic BP (mm of Hg)	86 ± 40	83 ± 9	83 ± 5	81 ± 5	83 ± 9	79 ± 9
IVSd (mm)	12 ± 3	13 ± 2	12 ± 2	12 ± 2	12 ± 2	12 ± 2
LVPWd (mm)	11 ± 3	11 ± 2	11 ± 2	10 ± 1	11 ± 1	11 ± 2
LVIDd (mm)	46 ± 5	42 ± 7*	53 ± 7	50 ± 7*	51 ± 7	47 ± 8*
LVEF (%)	53 ± 13	54 ± 13	49 ± 13	48 ± 13	43 ± 11	46 ± 10
Mitral E (cm/s)	130 ± 32	103 ± 28^†^	116 ± 32	105 ± 26	127 ± 23	84 ± 20*
Mitral A (cm/s)	99 ± 45	102 ± 34	79 ± 33	94 ± 38	93 ± 21	101 ± 26
E/A Ratio	1.5 ± 0.5	1.1 ± 0.4^†^	1.8 ± 1	1.4 ± 1^‡^	1.5 ± 0.6	0.9 ± 0.2^‡^
DT (ms)	108 ± 34	116 ± 42	115 ± 28	116 ± 34	131 ± 41	177 ± 60^†^

Data are means ± SD. Within group comparisons were made using paired 't' test. * p < 0.05;^† ^p < 0.01;^‡^p < 0.001. IVSd = Interventricular septum in diastole; PWDd = Posterior wall thickness in diastole; LVIDd = Left ventricular internal diameter in diastole; DT = Decelaration time of E velocity. LVEF= Left ventricular ejection fraction.

Table 4 (Pre-HD, upper panel) shows results of ANOVA with between- groups comparisons of LV regional data pre- and post- HD. At pre-HD there were no significant differences of PSV in the septum among the patient groups, but in the other LV walls PSV was significantly higher in the ESRD group than in the other two groups (all p < 0.05). E' velocity in the ESRD group was higher only in the anterior and the inferior walls when compared with the other groups. A' velocity in the ESRD group was higher only in the lateral wall in comparison with the other groups. LV displacement at predialysis was higher in ESRD group in the inferior wall when compared with ESRD+DM+CAD group and in the anterior wall it was higher compared with the other 2 groups (all p < 0.05).

**Table 4:** Comparisons of the left ventricular regional TVE parameters pre- (upper panel) and post- HD (lower panel). Data are mean ± SD

**Groups & LV bases**	**PSV**	**E' cm/s**	**A' cm/s**	**Disp.mm**
**ESRD**				
Septum	5.7 ± 1.6	-5.7 ± 1.9	-6.4 ± 3.5	8.9 ± 3.2
Lateral	5.9 ± 2.7*	-8.4 ± 3.6	-10.4 ± 4.0^§^	8.1 ± 3.7
Inferior	6.6 ± 1.4^†^	-8.9 ± 3.5^#¶^	-5.9 ± 2.3	11.2 ± 3.3**
Anterior	7.1 ± 1.9^‡^	-8.4 ± 3.3^#¶^	-5.4 ± 3.1	9.4 ± 2.3^††^
**ESRD+DM**				
Septum	4.7 ± 1.5	-5.1 ± 1.5	-5.6 ± 3.1	7.4 ± 2.2
Lateral	4.5 ± 1.4	-6.3 ± 2.1	-5.0 ± 3.3	5.7 ± 2.6
Inferior	4.9 ± 1.1	-5.4 ± 1.8	-7.1 ± 3.0	9.0 ± 3.5
Anterior	4.8 ± 1.4	-4.4 ± 1.6	-5.2 ± 2.9	6.5 ± 2.7
**ESRD+DM+CAD**				
Septum	4.6 ± 1.1	-5.5 ± 1.5	-6.0 ± 2.6	6.8 ± 2.4
Lateral	3.9 ± 1.1	-6.5 ± 2.5	-3.6 ± 1.6	5.4 ± 2.2
Inferior	4.9 ± 1.4	-5.9 ± 1.3	-7.1 ± 2.4	8.2 ± 2.3
Anterior	4.4 ± 1.1	-5.9 ± 1.9	-4.9 ± 3.3	6.3 ± 1.7
**ESRD**	***Post Hemodialysis data***			
Septum	7.2 ± 2.3*^†^	-7.5 ± 2.1^‡#^	-8.9 ± 3.5^¶^	7.7 ± 3.4^§^
Lateral	7.8 ± 3.8	-10.2 ± 4.0	-11.5 ± 4.0	7.6 ± 3.8
Inferior	7.9 ± 2.1	-9.9 ± 3.4	-8.1 ± 3.2	8.8 ± 3.6
Anterior	8.6 ± 2.1	-9.9 ± 3.3	-7.7 ± 3.9	8.0 ± 2.9
**ESRD+DM **				
Septum	5.2 ± 1.3	-4.6 ± 1.0	-8.1 ± 3.1	7.0 ± 2.4
Lateral	4.9 ± 1.3	-6.6 ± 2.5	-6.5 ± 4.2	6.2 ± 2.7
Inferior	5.2 ± 1.6	-5.5 ± 2.7	-8.3 ± 3.1	9.2 ± 3.8
Anterior	5.0 ± 1.5	-4.9 ± 1.7	-6.13 ± 3.1	5.7 ± 1.9
**ESRD+DM+CAD**				
Septum	4.1 ± 1.3	-3.8 ± 2.5	-5.5 ± 2.1	4.7 ± 1.8
Lateral	4.3 ± 1.7	-5.1 ± 2.3	-3.5 ± 2.2	5.8 ± 2.4
Inferior	4.6 ± 1.1	-4.1 ± 2.1	-7.8 ± 2.4	7.1 ± 2.6
Anterior	4.9 ± 1.3	-3.9 ± 2.4	-5.7 ± 2.4	5.6 ± 2.3

Pre-HD comparisons: *p < 0.05, ^† ^p < 0.01, ^†† ^p < 0.001,^§ ^p < 0.001,^††^p < 0.01 vs. other 2 groups.#p < 0.001 vs. Group 2. ¶p < 0.05 vs. Group3. **p < 0.05 vs. ESRD+DM+CAD. Post-HD comparisons: * p < 0.05 vs. Group 2 and Group 3; ^† ^p < 0.01 vs. Group 2 and Group 3; ^‡ ^p < 0.01 vs. Group 2 and Group 3 ; #p < 0.001 vs. Group 2 and Group 3; ¶p < 0.05 vs. Group3; ^§ ^p < 0.05 vs. Group 3 (all comparisons in septum); PSV = peak myocardial systolic velocity; E' = early myocardial diastolic velocity; A' = late myocardial diastolic velocity; Disp = displacement. Group comparisons (pre- and post- HD) were made using one- way ANOVA followed by Scheffe's test.

Following HD (Table 4, lower panel), the PSV and E' were significantly higher in the ESRD group compared with the other 2 groups, while A' and longitudinal systolic displacement in the ESRD group were significantly higher in ESRD group compared with ESRD +DM+CAD in the LV septum.

**Figure 2:**
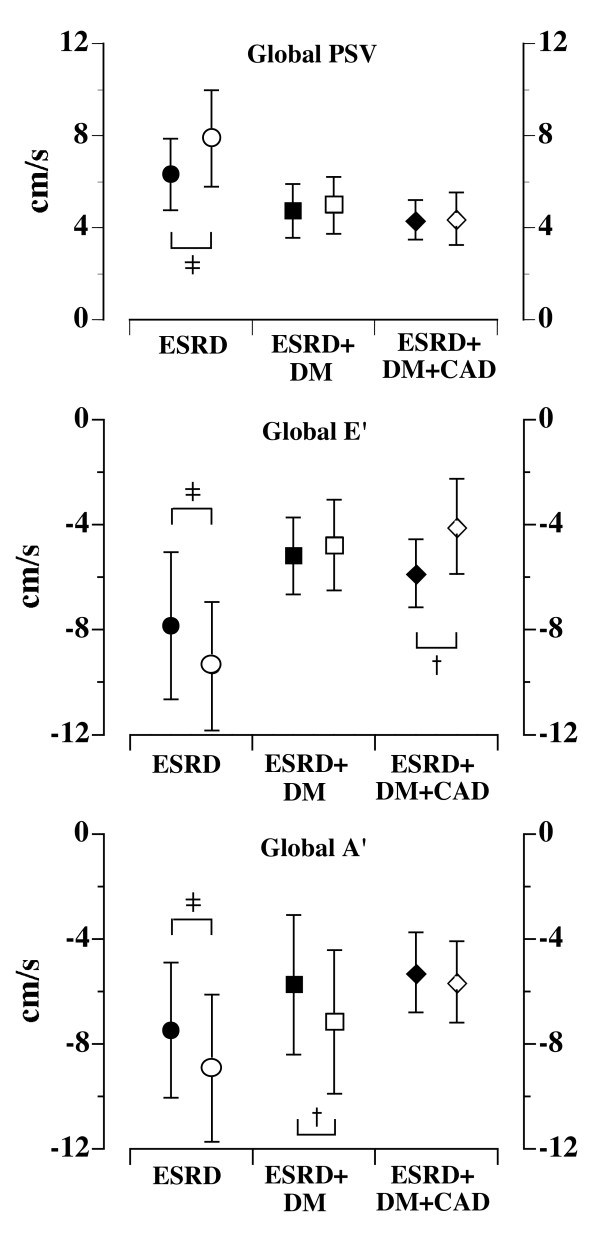
Global longitudinal myocardial systolic (PSV), early (E') and late diastolic (A') velocities, pre- and post-hemodialysis. † p < 0.01; ‡ p < 0.001. Results were obtained by taking average of 4 left ventricular basal segments pre- (closed symbols) and post- (open symbols) hemodialysis. Data are mean ± SD

Figure 2 shows comparisons of pre- and post-HD effects on global LV systolic and diastolic functions. The global PSV was significantly increased in the ESRD group after dialysis but remained unchanged in the other two groups. The global E' velocity was increased in ESRD group, but decreased in ESRD+DM+CAD group while the global A' velocity was increased in the ESRD and ESRD+DM groups.

**Figure 3:**
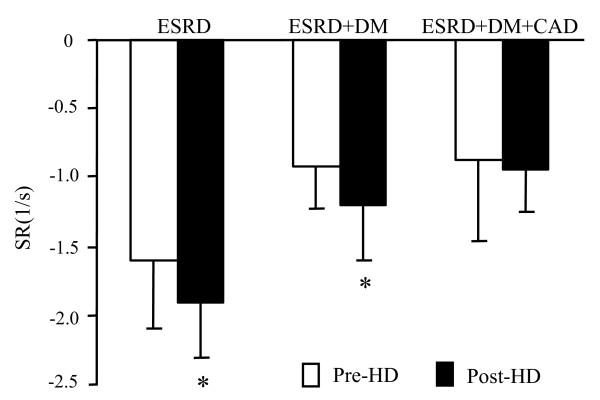
Global longitudinal strain rate measured pre- and post-hemodialysis. * p < 0.001 for within-group paired comparisons between pre-and post-HD. Data represent average values of strain rate measurements obtained from the 12 left ventricular segments.

Figure 3 shows global LV strain rate pre- and post-HD. While subjects with ESRD as well as those with only DM had increased strain rate following dialysis indicating increased myocardial deformation velocity, the ESRD+DM+CAD group did not show any significant change.

### Radial TVE data

Radial images at pre-HD did not show any significant difference among the groups. Post-HD measurements however revealed PSV, E' velocity and SR increased in the ESRD group, as they were seen longitudinally. While only A' velocity was increased in ESRD+DM group, PSV and SR were increased in the ESRD+DM+CAD group (data not shown). A significant decrease in IVCT (79.50 ± 29.14 msec vs. 63.14 ± 16.39 msec, p < 0.05) after HD was observed in the latter group as well. E'/A' ratio was significantly lower in all the groups. Ejection, isovolumic relaxation, and filling times as well as LV displacement showed no significant differences.

### Systolic and diastolic time intervals, LV filling variables and Tei index

Measurement of IVCT, ET, IVRT and FT at pre- HD did not differ. ESRD group at post- HD showed global and regional decrease of ejection time (ET) in all the LV walls (data not shown). In the ESRD+DM group filling time was decreased only in septum, while in the ESRD+DM+CAD group there was global and regional decrease in ET in septum, lateral and anterior wall. Isovolumic relaxation time (IVRT) was however prolonged globally after HD (73.55 ± 9.87 msec vs. 101.42 ± 19.55 msec; p < 0.001) in the same group. On the other hand, isovolumic contraction time (IVCT) did not show any significant changes.

LV filling pressures were assessed indirectly by calculating E/E' ratio at the septal and lateral annulus, pre- and post-HD. At pre-HD, there were no significant differences among the groups (data not shown). There was however a decrease in E/E' ratio at post-HD only in the ESRD group when measured separately and also when the average of the two walls was done (23.28 ± 15.85 vs. 14.42 ± 8.84; p < 0.05).

LV Global Tei index was significantly higher after HD (0.67 ± 0.22) than before (0.57 ± 0.10 vs.;p < 0.05) in Group 1 and in Group 3 (0.72 ± 015 vs. 0.54 ± 0.10; p < 0.001), while remained unchanged in Group 2 (0.72 ± 0.15 vs. 0.61 ± 0.14.; p = ns).

## Discussion

The principal findings of the study are: firstly, global left ventricular longitudinal systolic and diastolic myocardial velocities and systolic strain rate become higher in ESRD group post-HD; secondly, these variables are either unchanged or even become worse (E' velocity in Group 3, vide Figure 2) in the other 2 groups; thirdly, radial velocities and strain rate also becomes higher in ESRD while minimal compensatory over-activities of the radial systolic functions (velocity and strain rate) are noted in the ESRD+DM+CAD groups. Finally, despite significant reduction in dialysis-dependent preload, LV filling pressures, as assessed by E/E' ratio, decreased only in ESRD group, indicating that acute changes in loading conditions influence the TVE variables in a different way depending on the background diseases.

The improved LV functions in ESRD groups in the present study confirm the findings reported by others [17,18] and could be explained by the fact that there is improved myocardial perfusion, which is not only because of removal of fluids, solutes and toxins [19], but also because of improvement in myocardial interstitial oedema [20]. Another reason that can be attributed to this improvement is that LV hypertrophy was not seen in this group as frequently as in the other 2 groups. A modest increase in Tei index was however observed in this group of patients post HD, suggesting somewhat decreased myocardial performance, not uncommonly seen even in even patients with isolated ESRD [21]. However, the absolute value was far below then the threshold of 0.91 seen in patients with heart failure [22].

A subtle form of diabetic heart muscle disease is recognizable by TVE at rest as has been shown by Fang ZY et al [10], possibly reflecting microvascular disease in the earliest form of diabetic cardiomyopathy [23], since stress velocity response was normal. Moreover, it has been reported that the patients with ESRD + DM and angiographically normal coronary arteries have a lower coronary flow reserve (CFR) than patients with DM alone [24]. Decreased lumen area, vascular remodeling and increased LV mass may explain this attenuated CFR. These mechanisms are often related to microvascular disease of DM, hypertensive heart disease, and CAD [25,26], and may have influenced the current post-HD data in the groups with concomitant DM and CAD. In addition, when diabetes is associated with significant coronary disease the LV myocardial velocities progressively decrease [11]. It is therefore not surprising that in patients with ESRD+DM+CAD had additional disturbances, such as prolonged IVRT, diminished systolic displacement, unchanged LV filing pressures and significantly higher Tei index post HD

## Conclusion

A single HD session improves LV functions in ESRD only in absence of co-morbidities. In presence of DM, myocardial functions continue to remain diminished irrespective of concomitant CAD. Minor compensatory over-activity of systolic radial motion can be seen in the latter group, possibly because of the general regional nature of CAD. Our data suggest that the observed HD-induced differences in TVE variables by and large depend on the preexistent myocardial functional status and associated hemodynamic milieu during the time of investigation.

### Study limitations

The younger age in the ESRD group could have influenced the increase in systolic and diastolic velocities and strain rate. However, published data from our group as well as those from others indicate that presence of DM and CAD has ominous effects on LV functions irrespective of age. We therefore believe that the age differences have not influenced the obtained results. We have studied only one randomly chosen HD session and hence it is difficult to reflect on the natural history of progression of LV dysfunction in these patients.

### Clinical implications

In patients undergoing maintenance HD the hemodynamic status is in a state of constant flux. There is no well-established technique to quantitatively monitor serial changes of myocardial functions in patients with ESRD undergoing periodic HD. However, the results of the present study have shown that in contrast to standard Doppler, TVE offers a unique choice to extensively study myocardial functional differences resulting from dialysis-dependant preload changes and the procedure may be of importance in following patients with associated DM and/or CAD in the setting of ESRD. At the same time the ESRD subjects may also offer a unique opportunity to study influence of loading conditions clinically by application of TVE methods that are more robust and perhaps less load- dependent than conventional Doppler methods at least within physiological limits.

## Competing interests

The author(s) declare that they have no competing interests.

## Authors' contributions

SCG performed all the echocardiographic investigations before as well as after hemodialysis and has been responsible for digital storage of the images and collected all the clinical data of the study subjects. SR did post-processing of the images along with SCG. LÅB was the scientific supervisor of the project and was responsible for final production of the manuscript. JN provided major scientific input into the study, reviewed the manuscript, drew Fig 2, and was mainly responsible for answering the queries of the referees along with SKS. SSR was the scientific supervisor of the study in India and has been in charge of the catheterization laboratory and performed coronary angiography. SKS participated in the design of the study, carried out all statistical analyses, produced first version of the manuscript and was responsible for creation of all the tables as well as Figures 1 and 3. SKS is the corresponding author. All authors read and approved the final manuscript.
